# KSHV-Encoded MicroRNAs: Lessons for Viral Cancer Pathogenesis and Emerging Concepts

**DOI:** 10.1155/2012/603961

**Published:** 2012-02-19

**Authors:** Zhiqiang Qin, Andrew Jakymiw, Victoria Findlay, Chris Parsons

**Affiliations:** ^1^Department of Medicine, Hollings Cancer Center, Medical University of South Carolina, 86 Jonathan Lucas St., Charleston, SC 29425, USA; ^2^Department of Craniofacial Biology, Medical University of South Carolina, 173 Ashley Avenue, Charleston, SC 29425, USA; ^3^Key Laboratory of Arrhythmias, Ministry of Education, and Research Center for Translational Medicine, Shanghai East Hospital, Tongji University School of Medicine, Shanghai 200120, China; ^4^Department of Pathology, Hollings Cancer Center, Medical University of South Carolina, 86 Jonathan Lucas St., Charleston, SC 29425, USA

## Abstract

The human genome contains microRNAs (miRNAs), small noncoding RNAs that orchestrate a number of physiologic processes through regulation of gene expression. Burgeoning evidence suggests that dysregulation of miRNAs may promote disease progression and cancer pathogenesis. Virus-encoded miRNAs, exhibiting unique molecular signatures and functions, have been increasingly recognized as contributors to viral cancer pathogenesis. A large segment of the existing knowledge in this area has been generated through characterization of miRNAs encoded by the human gamma-herpesviruses, including the Kaposi's sarcoma-associated herpesvirus (KSHV). Recent studies focusing on KSHV miRNAs have led to a better understanding of viral miRNA expression in human tumors, the identification of novel pathologic check points regulated by viral miRNAs, and new insights for viral miRNA interactions with cellular (“human”) miRNAs. Elucidating the functional effects of inhibiting KSHV miRNAs has also provided a foundation for further translational efforts and consideration of clinical applications. This paper summarizes recent literature outlining mechanisms for KSHV miRNA regulation of cellular function and cancer-associated pathogenesis, as well as implications for interactions between KSHV and human miRNAs that may facilitate cancer progression. Finally, insights are offered for the clinical feasibility of targeting miRNAs as a therapeutic approach for viral cancers.

## 1. Introduction

MicroRNAs (miRNAs) are small (19–24 nucleotides in length), noncoding RNAs that bind both untranslated and coding regions of target mRNAs, marking them for degradation or posttranscriptional modification. The biogenesis of miRNAs begins in the nucleus where RNA polymerase II generates primary miRNA (pri-miRNA) transcripts. Subsequently, pri-miRNAs are processed by the RNase III enzyme Drosha, generating precursor miRNAs (pre-miRNAs). Nuclear pre-miRNAs are then transported to the cytoplasm by exportin/Ran-GTP where they are cleaved by the cytoplasmic RNase III enzyme Dicer, generating mature miRNAs which are incorporated into the RNA-induced silencing complex (RISC). This directs RISC to the target mRNA based on sequence complementarity, resulting in gene silencing [1, 2]. miRNAs are encoded by many different organisms and regulate a variety of cellular processes, including cell proliferation, apoptosis, differentiation, and development [3].

Viruses encode miRNAs whose sequences and functions are unique from human miRNAs and miRNAs encoded by human herpesviruses have been increasingly well characterized [4]. Herpesviruses are enveloped, double-stranded DNA viruses, and the human gamma-herpesviruses, Epstein-Barr virus (EBV) and Kaposi's sarcoma-associated herpesviruses (KSHV), are the etiologic agents of several forms of cancer. As with other herpesviruses, the KSHV lifecycle involves two distinct phases: latent and lytic. During latency (the predominant phase in the majority of infected cells) only a limited number of viral genes are expressed. Provocation by a variety of stimuli induces lytic replication, resulting in virion assembly and release of infectious viral particles[5]. Existing data suggest that the oncogenic potential of KSHV is largely dependent upon genes expressed during latency, although “low level” replication occurring in a small minority of cells is also critical for infection of naïve cell targets, maintenance of the KSHV reservoir, and tumor pathogenesis [6–8]. Cancers caused by KSHV, including multicentric Castleman's disease (MCD), primary effusion lymphoma (PEL), and Kaposi's sarcoma (KS), arise preferentially in the setting of immune suppression as seen with HIV infection and provision of immunosuppressive medications in the context of solid organ transplantation [9–11].

Thus far, 12 KSHV pre-miRNAs, encoding 18 mature miRNAs, have been identified [12–14]. Within the KSHV genome, miRNAs are located in the KSHV latency-associated region (KLAR). Other proteins encoded within the KLAR are critical for maintenance of the viral episome and KSHV oncogenesis, including the latency-associated nuclear antigen (LANA), virus-encoded Cyclin (vCyclin), viral FLICE inhibitory protein (vFLIP), and kaposin (K12). 10 of 12 miRNAs (miR-K12-1~9 and 11) are located within the intron of K12; miR-K12-10 is located within the open reading frame of K12 A/C and the 3′UTR of K12 B, and miR-K12-12 is located within the 3′UTR of K12 [12–14]. Given their location within the KLAR, it follows that KSHV miRNAs facilitate maintenance of latent viral gene expression and the oncogenic potential of these genes. This paper will summarize recent findings regarding the expression of KSHV miRNAs and their regulatory functions and elaborate on emerging mechanistic concepts in this field. We will also review several recently published studies offering insight into the feasibility of targeting miRNAs for therapeutic purposes. For an overview of KSHV miRNA targets and their putative functions, see Table 1. 

## 2. Expression Patterns for KSHV miRNAs

Expression of KSHV miRNAs has been demonstrated within latently infected primary human cells and KSHV-infected PEL cells [12–16]. PEL cell lines exhibit significant conservation (~99.6%) of KSHV-miRNA expression [17], although one group recently reported that miR-K12-9 may be mutationally inactivated in different PEL lines [18]. Moreover, expression levels for individual KSHV miRNAs vary considerably [13]. Phylogenetic analyses of KSHV miRNA sequences from clinical samples of KS and MCD patients of divergent geographic backgrounds reveal the existence of 2 major sequence clusters, referred to as the major (A/C) and variant (B/Q) clusters [17]. Further analyses of the pre-miRNA sequences show that some KSHV miRNAs are highly conserved (such as miR-K12-1, 3, 8, 10, 11, and 12), while others (including miR-K12-2, 4, 5, 6, 7, and 9) exhibit sequence alterations likely affecting their processing and function, although this hypothesis requires additional confirmation [17]. In addition, one study found distinct polymorphisms within pri-miRNAs, pre-miRNAs, or mature miRNAs encoded by circulating KSHV in a European patient cohort, and some of these polymorphisms may affect mature miRNA processing and associate with KS risk [19]. Collectively, these data indicate that individual KSHV miRNAs may regulate KSHV pathogenesis in a disease-specific manner, and that they may exhibit cell type-specific functions within the tumor microenvironment. Identification of viral and cellular factors governing these differences should illuminate additional mechanisms and help determine whether screening for miRNA polymorphisms can be used to quantify one's risk for developing KSHV-associated tumors.

## 3. KSHV miRNA Regulation of Virus Entry

We have reported that miR-K12-1, 9, and 11 increase macrophage and endothelial cell (EC) susceptibility to KSHV entry and latent gene expression through upregulation of xCT [20], an inducible amino acid exchanger and fusion-entry receptor for the virus [21]. One mechanism for these observations involves upregulation of xCT through miR-K12-11 repression of BACH-1, a negative transcription regulator of xCT [20]. These findings are consistent with earlier reports revealing direct targeting of BACH-1 by miR-K12-11 [22, 23]. Mechanisms for regulation of xCT expression by miR-K12-1 and 9 have not yet been elucidated. One related report noted involvement of KSHV miRNAs in endothelial cell reprogramming through repression of the cellular transcription factor Maf (cMaf) [24]. cMaf also serves as a negative transcription regulator for xCT, so we have speculated that multiple KSHV miRNAs, through cooperative mechanisms, facilitate KSHV entry [20]. Whether KSHV miRNAs regulate expression and/or function of other cellular receptors for KSHV, including DC-SIGN and integrins [25–29], has not been established. Increased cell permissiveness for KSHV entry following initial infection and miRNA expression may represent an evolutionary mechanism for KSHV promotion of its own persistence. Supporting this hypothesis, several reports have shown that a significant proportion of KSHV-infected tumor cells contain multiple viral clones [30–32]. Moreover, downregulation of MHC Class I (MHC-I) in KSHV-infected cells is directly proportional to intracellular KSHV episome copy number [33], implying that an increase in intracellular viral copies reduces KSHV epitope presentation to CD8^+^T cells. In addition, precedence exists for human miRNA regulation of virus entry. For example, one group has demonstrated that several human miRNAs regulate monocyte/macrophage susceptibility to HIV infection [34, 35]. Another group reported that miR-23b inhibits Rhinoviruses 1B (RV1B) entry through targeting of the very low density lipoprotein receptor [36]. Therefore, it is plausible that KSHV and human miRNAs cooperatively regulate surface determinants of cell targeting by KSHV and other viruses. Furthermore, it is likely that KSHV and other viral miRNAs regulate secretion of microenvironmental factors by infected cells that influence susceptibility of neighboring cells to virus entry. We have shown that KSHV miRNA induce secretion of reactive nitrogen species (RNS), and that inhibition of the enzymatic generation of RNS reduces cell susceptibility to KSHV infection [20]. Given that both BACH-1 and cMaf are negative transcription regulators for genes containing antioxidant response elements (AREs) in their promoters, and since several genes involved in production of reactive nitrogen- and oxygen-based species (RNS and ROS, resp.) contain AREs, we hypothesize that KSHV miRNA regulation of BACH-1 and cMaf influences a more complex network of genes to generate tumor-promoting RNS and ROS while simultaneously protecting KSHV-infected cells from oxidative damage inflicted by these species [20]. These data have implications for development of therapeutic strategies to reduce KSHV infection in the tumor microenvironment and, therefore, KS progression [6–8].

## 4. KSHV miRNA Regulation of Viral Gene Expression

Maintenance of latent KSHV infection, coordinated with lytic reactivation within a small subset of infected cells, is critical for simultaneous promotion of KSHV persistence and dissemination. Studies published recently indicate a role for KSHV miRNAs in the regulation of this latent-lytic “switch”. miR-K12-7 and 9 bind and repress transcription of the KSHV immediate-early gene ORF50 which encodes the replication and transcription activator (RTA) [37, 38]. RTA activation is critical for the initiation of lytic replication of the virus [37, 38]. miR-K12-4 represses expression of the retinoblastoma (Rb)-like protein 2 (Rbl2), a known repressor of DNA methyl transferases (DNMT)-1, -3a and -3b. Increased activity of these DNMTs maintains methylation of the RTA promoter and suppresses its expression [40]. Furthermore, miR-K12-1 targeting of I*κ*B*α*, an inhibitor of NF-*κ*B complexes, promotes NF-*κ*B-dependent viral latency and cell survival [41]. miR-K12-3 also promotes KSHV latency through targeting of nuclear factor I/B, an activator of the RTA promoter [42]. Conversely, miR-K12-5 and 9 repress the Bcl-2-associated factor (BCLAF1), resulting in an increase in lytic replication, albeit through mechanisms that have yet to be defined [39]. One recent study indicates that miR-K12-11 targets and downregulates IKK*ɛ*, a signaling intermediate shown previously to facilitate lytic reactivation of KSHV from latently infected cells [43]. Another report revealed upregulation of two miRNAs, miR-K12-10, and 12, during chemical induction of KSHV lytic reactivation [18], but whether these miRNAs play an active role in regulation of the lytic switch for KSHV remains to be determined. Collectively, these data support the notion that KSHV miRNAs function primarily to maintain viral latency, congruous with their location within the KLAR. This is also supported by recent work revealing that cells infected with KSHV deletion mutants lacking KSHV miRNAs exhibit increased expression of lytic viral genes, including ORF50 [40, 41].

## 5. KSHV miRNA Regulation of Cytokine Responses, Immune Recognition, and Cell Survival

Several factors secreted by KSHV-infected cells (and other cells found within the tumor microenvironment), including VEGF, IL-8, IL-6, IL-10, IL-1*β*, and TNF-*α*, support KSHV-associated pathogenesis through complimentary mechanisms involving interference or augmentation of cellular functions relevant to cancer pathogenesis [49]. More specifically, IL-6 and IL-10 collectively promote growth of KSHV-infected tumor cells, angiogenesis and suppression of T-cell activation [50–53]. We have demonstrated that KSHV-miRNAs induce IL-6 and IL-10 secretion by murine macrophages and human myelomonocytic cells, and that this is accomplished, in part, through miR-K12-3 and 7 repression of a dominant-negative isoform of C/EBP*β* which serves as a transcriptional repressor of IL-6 and IL-10 [44]. However, it remains unclear whether this effect results from direct targeting of C/EBP*β* by these miRNAs or an indirect effect within a more complex regulatory network. Furthermore, whether these events occur following *de novo* infection of human primary cells is unknown. A more recent study reported that miR-K12-11 induces splenic B-cell expansion and KSHV-associated lymphomagenesis through direct targeting of C/EBP*β* [45]. It is plausible that lymphomagenesis in this model is dependent on miRNA regulation of cytokine responses through targeting of C/EBP*β*, congruous with existing clinical data suggesting a role for cytokines in PEL pathogenesis [54]. Another study reveals that KSHV-encoded ORF57 competes with human miRNAs for binding of transcripts for both human IL-6 (hIL-6) and the KSHV-encoded viral homolog for IL-6 (vIL-6) [55]. In doing so, ORF57 impairs hIL-6 and vIL-6 RNA association with human miRNA-specified RISCs, thereby stabilizing IL-6 RNA.

Existing data further indicate that KSHV miRNAs may facilitate conditional *suppression* of cytokine responses and immune recognition. miR-K12-10 repression of the tumor necrosis factor-like weak inducer of apoptosis receptor (TWEAKR) in primary human ECs results in decreased expression of IL-8 and monocyte chemoattractant protein 1 (MCP-1) which are normally induced following TWEAKR interactions with its cognate ligand, TWEAK [46]. In addition, one group has found that KSHV miRNAs repress expression of the stress-induced natural killer (NK) cell ligand, MICB, thereby permitting escape of KSHV-infected cells from NK cell recognition and killing [47]. It seems likely that KSHV miRNA regulation of cytokine responses and immune evasion is a finely coordinated effort hinging on intracellular and/or exogenous microenvironmental signals that are cell type-specific. 

Maintenance of viability for KSHV-infected cells depends, in part, on KSHV regulation of cellular pathways promoting cell survival and antiapoptotic signaling. Several studies indicate that KSHV miRNAs are involved in this process. Microarray analyses using cells stably expressing KSHV-encoded miRNAs revealed that 3′UTRs of select cell proliferation/apoptosis-associated genes, including SPP1, S100A2, and PRG1, are likely targeted by multiple KSHV miRNAs [56]. However to our knowledge, specific target sequences within the 3′UTRs for these genes have not yet been validated, and functional correlates for KSHV miRNAs targeting these genes have not been determined. As mentioned previously, miR-K12-10 represses TWEAKR, and cells transfected with miR-K12-10 are more resistant to TWEAK-induced apoptosis [46]. Another group showed that expression of the cellular cyclin-dependent kinase inhibitor p21, a key inducer of cell cycle arrest, is repressed through its direct targeting by miR-K12-1 [48]. Ectopically expressed miR-K12-1 strongly attenuated cell cycle arrest induced during p53 activation through repression of endogenous p21. In summary, KSHV miRNA support of anti-apoptotic signaling, coupled with their regulation of cytokine responses and their putative role in suppression of immune recognition, suggests that KSHV miRNAs invoke cooperative mechanisms critical for survival of KSHV-infected cells.

## 6. Future Directions

### 6.1. Establishing Biologic Assays for Identification of KSHV miRNAs Targets

Online miRNA databases (http://www.mirbase.org/) and bioinformatics programs have been developed to predict virus-encoded miRNA targets [22, 57–59]. Several groups have utilized these programs for identifying putative targets of KSHV miRNA [14, 20, 22, 24, 40, 44], although the use of seed sequence matching as the principal predictive tool for these programs is too stringent given that many valid targets of miRNAs will not meet predetermined sequence matching criteria [60]. This has led to interest in developing screening tools involving more direct assessment of viral miRNA regulation of potential targets. One group published their use of a tandem array-based screening approach: first, they quantified expression of host genes under conditions of either KSHV miRNA overexpression or inhibition of single KSHV miRNAs in latently infected cells; second, they identified targets using stringent criteria including seed sequence complementarity at positions 2–8 which, although not required for targeting, has been associated with more reliable prediction of target downregulation [39]. Through this effort, they identified one gene targeted by miR-K12-5 (BCLAF1). As noted by the authors, limitations for this approach are its labor-intensive nature and lack of all-inclusiveness in target identification. Another group performed immunoprecipitation of RISCs followed by microarray analysis of the RISC-bound miRNA targets (RIP-Chip) of KSHV miRNAs, EBV-miRNAs, and human miRNAs using latently infected or stably transduced human B-cell lines [61]. Two targets were validated for EBV miRNAs, and transcript half-life of human and viral miRNA targets correlated inversely with recruitment to RISC complexes, indicating that RIP-Chip may offer a quantitative estimate of viral miRNA function [61]. Furthermore, two putative targets exhibited miRNA binding sites within their coding sequences, not within 3′UTRs. Additional studies should clarify whether these and other methods are ultimately cost-effective and yield more reliable identification of viral miRNA targets relative to bioinformatics screens. 

### 6.2. KSHV Regulation of Human miRNAs

Although the majority of published work has thus far focused on defining KSHV miRNA targets and functional correlations, data published more recently also suggest that KSHV-encoded proteins regulate cellular machinery by virtue of their regulation or interference with cellular miRNA functioning. In KS and PEL tumors, tumor-suppressor miRNAs, including miR-221, miR-222, and let-7 family members, are underrepresented [62]. Furthermore, pre-miRNA signatures may define the stages of EC transformation following KSHV infection [63]. More specifically, the loss of miR-221 expression marks the transition from immortalization to tumorigenicity for these cells [63]. Since the publication of these studies, several groups have identified specific mechanisms for KSHV regulation of cellular miRNAs. KSHV-encoded vFLIP represses expression of the chemokine receptor CXCR4 through NF-*κ*B-mediated upregulation of miR-146a [64]. Since KSHV encodes redundant mechanisms for NF-*κ*B upregulation, and since multiple cellular miRNAs have NF-*κ*B binding sites within their promoters, this study illuminates an important mechanism for KSHV regulation of the cellular miRNA machinery. Another elegant study recently confirmed that KSHV induces EC migration through regulation of cellular transcription factors, and the authors identified two complimentary mechanisms for this effect [65]. First, they found that the transcription factors ETS2 and ETS1 are downstream targets of cellular miR-221 and miR-222, respectively. They confirmed that two KSHV-encoded latent proteins, LANA, and Kaposin B, downregulate the miR-221/miR-222 cluster through direct interactions with the miR-221/miR-222 promoter resulting in upregulation of ETS1/2-induced EC mobility [65]. Second, they found that KSHV upregulates EC expression of miR-31, thereby repressing expression of the tumor suppressor FAT4. They confirmed the presence of miR-31 binding sites within the coding region of FAT4, and that KSHV/miR-31-induced suppression of FAT4 results in enhanced EC mobility. The same group published additional data suggesting that the minor variant of KSHV-encoded K15 induces cell migration and invasion through induction of miR-31 [66]. KSHV regulation of cellular miRNAs may also influence immune evasion and immunopathogenesis. KSHV infection induces expression of miR-132, thereby reducing expression of interferon (IFN)-stimulated genes and facilitating viral replication in EC [67]. And as previously mentioned, KSHV-encoded ORF57 competes with cellular miRNAs for binding of transcripts for IL-6, thereby stabilizing IL-6 RNA [55]. These studies further underscore the complex regulatory network of viral and human miRNAs that contribute to tumor pathogenesis, and future studies will confirm whether inhibition of KSHV regulation of human miRNAs offers a viable therapeutic strategy for KSHV-associated diseases.

### 6.3. Regulation of KSHV miRNA Expression

Numerous studies have focused on defining the regulatory functions of miRNAs. Less well understood are mechanisms for transcriptional and posttranscriptional regulation of miRNAs themselves, including viral miRNAs, although burgeoning data suggest that these processes are important for cancer pathogenesis [68, 69]. miRNAs are under the control of a wide range of transcription factors, including some tumor suppressors and oncogenes [70–72], and recent reports reveal that certain environmental conditions like hypoxic stress influence miRNA expression. miR-210 is induced by hypoxia-inducible factor-1 alpha (HIF-1*α*) to promote cell survival and adaptation to hypoxic environmental conditions [73], and HIF-1*α* alters miR-101 expression in a prostate cancer model [74]. Interestingly, HIF-1*α* is highly expressed in HIV-associated KS lesions [75], and KSHV-encoded IFN regulatory factor 3 (vIRF3) stabilizes HIF-1*α*, thereby inducing vascular endothelial growth factor (VEGF) expression [76]. KSHV-encoded LANA also functions both as an inhibitor of a HIF-1*α* suppressor, the von Hippel-Lindau protein, and as an inducer of nuclear accumulation of HIF-1*α* during latent KSHV infection [77, 78]. These data would support additional work to determine whether KSHV regulation of HIF-1*α* dysregulates human miRNA expression and tumor pathogenesis. Other factors regulate miRNA expression at the posttranscriptional level, including Drosha and its interactional protein DGCR8 [79–81]. One study also noted that a single nucleotide polymorphism within the miR-K12-5 precursor stem-loop reduces Drosha processing and inhibits mature miR-K12-5 expression in BCBL-1 cells [82]. This implies that mutations within miRNA genes themselves may arise during the transformation of an infected cell and differential expression of KSHV miRNAs which favor specific pathogenic events. DNA methylation and histone deacetylation also contribute to regulation of miRNA transcription [83–85]. A report referenced previously found that genomic DNA from cells infected with a KSHV deletion mutant lacking 10 of the 12 mature KSHV miRNAs exhibited a striking loss of methylation [40], but whether miRNA expression is regulated through epigenetic mechanisms, possibly involving miRNAs themselves, has not been elucidated.

### 6.4. Targeting Viral and Cellular miRNAs for Clinical Applications

Despite challenges in achieving efficient and selective approaches for suppressing miRNA functions *in vivo*, the concept of targeting miRNAs for therapeutic benefit has gained considerable attention with the publication of elegant studies revealing effective methods for suppressing miRNA-associated tumor progression in animal models. One of the first examples of chemical modification of oligonucleotides for miRNA inhibition was the development of antagomirs, small ribonucleotide chains whose 2′-hydroxyl on the ribose is replaced by a 2′-O-Methyl group for stability [86]. Commercially available antagomirs have additional modifications that stabilize miRNA-antagomir binding and prevent recognition of cognate mRNAs by miRNAs. Antagomirs have demonstrated utility for inhibiting KSHV miRNA-induced pathogenesis in KSHV-infected cells *in vitro *[20, 44, 56]. Intravenous delivery of antagomirs has also demonstrated utility *in vivo *[86–88], but off-target effects and excessive doses required to suppress miRNA expression have raised concerns about the utility of this approach [89]. 

Examples of other chemical modifications of oligonucleotides for clinical applications include morpholinos and locked nucleic acids (LNAs). Morpholinos contain six-member morpholine rings rather than five-member ribose rings, conferring resistance to nucleases [90]. Morpholinos may be further engineered to bind and protect mRNA target sequences from miRNA to confer superior target specificity [91]. Morpholinos conjugated to peptides to enhance cell penetration have demonstrated utility in animal models [92], and a modified drug based on this technology, delivered by intramuscular injection, is undergoing evaluation in one clinical trial [93]. LNAs contain a biochemical modification where the 2′-oxygen and 4′-carbon atoms of the ribose rings are chemically bridged. This “locked” confirmation confers high thermal stability and resistance to exo- and endonucleases. An LNA-based miR-122 inhibitor is under evaluation for the treatment of hepatitis C [94]. In fact, LNAs have demonstrated utility in a number of animal model systems [95–99]. As noted previously, miR-K12-11, a KSHV-encoded ortholog of cellular miR-155, targets C/EBP*β*, [45], and one study indicates that silencing of miR-155 in mice using LNAs leads to derepression of C/EBP*β* [99]. It is interesting to speculate whether LNAs could be used to suppress KSHV-associated lymphoma progression *in vivo* using this approach to target miR-K12-11. 

As we discussed previously, KSHV itself suppresses expression of human miRNAs serving as tumor suppressors, including miR-221 and miR-222. This raises the question of whether delivery of selected miRNAs would interfere with KSHV pathogenesis *in vivo*. The utility of miRNA delivery for cancer therapeutics has been supported recently through studies indicating successful suppression of tumors *in vivo* using liposomal nanoparticles containing miRNA or lipid-based delivery reagents which are commercially available [100, 101]. In one study, systemic delivery of miR-34a inhibited prostate cancer metastasis and extended survival of tumor-bearing mice, in part through targeting of CD44 [101]. CD44 is one of two well-characterized receptors for hyaluronic acid (HA) [102], and we have recently reported effective sensitization of human KSHV-infected lymphoma cells to chemotherapy using various approaches for interfering with HA-receptor interactions [103]. Whether KSHV regulation of human miRNAs initiates upregulation of HA receptors and drug resistance for KSHV-infected cells remains unknown. Regardless, this reinforces the complex interplay between viral and human miRNA and the redundancy of viral miRNA regulatory mechanisms, and implies that viral cancer treatment approaches targeting a single miRNA would likely be limited in their clinical efficacy. Combining miRNA targeting with existing therapies for viral tumors may be a more tractable approach. Of note, *in vivo* effects of targeting multiple miRNAs simultaneously have not been defined, although simultaneous use of multiple antagomirs targeting KSHV miRNAs demonstrates additive or synergistic suppression of KSHV pathogenesis *in vitro *[20, 44, 56].

## 7. Conclusion

As the etiologic agent of diverse forms of human cancer and by virtue of its tropism for a variety of human cell types, KSHV represents a model pathogen for the study of viral miRNA expression and function. Elegant studies performed recently underscore the importance of KSHV miRNAs and their interactions with human miRNA for cancer pathogenesis, including viral biology and gene expression, cytokine responses, immune evasion, and anti-apoptotic signaling. The plasticity of these interactions and challenges inherent to miRNA targeting *in vivo *incur substantial obstacles for development of miRNA-based therapies, but recent advances hold considerable promise for eventual clinical application of therapeutic approaches targeting viral miRNAs in the treatment of viral malignancies.

## Figures and Tables

**Table 1:** Overview of KSHV miRNAs regulatory functions and targets.

Functions	KSHV miRNAs	Validated targets	References
KSHV entry	miR-K12-1	—	[20]
	miR-K12-9	—	[20]
	miR-K12-11	BACH-1	[20, 22, 23]

Induction of reactive nitrogen species (RNS)	miR-K12-1, 9 and 11	—	[20]

Endothelial cell reprogramming	miR-K12-6 and 11	MAF	[24]

KSHV gene expression	miR-K12-7	RTA	[37]
	miR-K12-9	RTA/BCLAF1	[38, 39]
	miR-K12-4	Rbl2	[40]
	miR-K12-1	I*κ*B*α*	[41]
	miR-K12-3	Nuclear factor I/B	[42]
	miR-K12-5	BCLAF1	[39]
	miR-K12-11	IKK*ε*	[43]
	miR-K12-10 and 12	—	[18]

Cytokine secretion	miR-K12-3 and 7	—	[44]
	miR-K12-11	C/EBP*β*	[45]
	miR-K12-10	TWEAKR	[46]

Immune escape	miR-K12-7	MICB	[47]

Cell survival	miR-K12-10	TWEAKR	[46]
	miR-K12-1	p21	[48]
